# The cumulative effect of ellagic acid and carnosic acid attenuates oxidative events during diabetic wound healing: in different applications and on different days

**DOI:** 10.55730/1300-0152.2712

**Published:** 2024-10-22

**Authors:** Elif Naz GÜRSOY, Kanuni Barbaros BALABANLI, Fatma Nur TUĞCU DEMİRÖZ, Şule COŞKUN CEVHER

**Affiliations:** 1 Department of Biology, Faculty of Science, Gazi University, Ankara, Turkiye; 2 Pharmaceutical Technology, Faculty of Pharmacy, Gazi University, Ankara, Turkiye

**Keywords:** Diabetic wound healing, carnosic acid, ellagic acid, matrix metalloproteinase, protein carbonyl

## Abstract

**Background/aim:**

The hyperglycemic environment in diabetes disrupts normal wound-healing processes, leading to chronic wounds. This study investigated whether the combination of the phenolic compounds ellagic acid and carnosic acid shows synergistic effects on diabetic wound healing and oxidative parameters in diabetic rats.

**Materials and methods:**

Diabetic rats were divided into control, untreated, Carbopol 974P treated, topical treatment, and oral gavage treatment groups. Ellagic acid and carnosic acid in combination were applied topically and as oral gavage to the full-thickness excisional wounds of all animals except for those in the control group. We investigated oxidative events with malondialdehyde, glutathione, nitric oxide, protein carbonyl, collagen spectrophotometrically, and matrix metalloproteinase 2 and 9 and advanced oxidation protein product levels using ELISA.

**Results:**

The combination of ellagic acid and carnosic acid decreased malondialdehyde, nitric oxide, protein carbonyl, advanced oxidation protein product, and metalloproteinase 9 levels and increased the rate of wound contraction and collagen levels in the diabetic wound healing model.

**Conclusion:**

The combined use of ellagic acid and carnosic acid showed a synergistic effect, enhanced wound healing, and decreased oxidative stress. This combination may provide effective therapy for chronic nonhealing wounds that occur as a complication of diabetes.

## 1. Introduction

Wound healing is the process of repairing impaired tissue integrity, involving a series of cellular, physiological, and biochemical events (Reinke and Sorg, 2012). Wound healing triggered by tissue destruction consists of four successive phases: hemostasis, inflammation, proliferation, and remodeling (Wang et al., 2018). Many local and systematic factors affect wound healing (Guo and Dipietro, 2010). Diabetes mellitus (DM), which is among the systematic factors and one of the most common diseases worldwide, is a complex metabolic disease. DM is characterized by insulin resistance, impaired insulin signaling, abnormal glucose and lipid metabolism, subclinical inflammation, and increasing oxidative stress (Salazar et al., 2016). Impaired wound healing and chronic wounds are among DM’s most essential complications (Domingueti et al., 2016). Diabetes-related nonhealing wounds can significantly impact a person’s life, both psychologically and economically. Therefore, many studies have been carried out on wound healing and wound care over the years (Burgess et al., 2021).

For thousands of years, natural remedies have been used as medicinal treatments. Nowadays, the use of natural medicinal plants as an alternative to medical treatments has increased considerably in the context of green chemistry. Due to the rising cases of chronic wound conditions, there is growing research interest in exploring the medicinal properties of natural plants to enhance wound healing. Plants have a remarkable ability to produce secondary metabolites such as phenolics, polyphenols, alkaloids, and terpenoids. Polyphenols, the most common secondary metabolites, are compounds found mainly in fruits, vegetables, and grains. Polyphenols formed by plants have properties such as antioxidant, antiallergic, antiinflammatory, and antimicrobial (Baek et al., 2016).

Ellagic acid is a member of flavonoids from polyphenolic compounds (C14H6O8; MW: 302.202; 2,3,7,8-tetrahydroxy [1] benzopyranol[5,4,3-cde] benzopyran-5,10-dione) and is naturally accessible in many fruits (pomegranates, blackberries, and raspberries), as well as in walnuts, almonds, and seeds (Derosa et al., 2016). Ellagic acid has been shown in different studies to have various biological properties such as antidiabetic, antitumor, antiinflammatory, and antioxidant activities (Priyadarsini et al., 2002; Baek et al., 2016; Wang et al., 2019).

Carnosic acid is a labdane-type phenolic diterpene found in plant species of the family Lamiaceae, such as rosemary (*Rosmarinus officinalis*) and sage (*Salvia officinalis*) (Birtić et al., 2015). Carnosic acid is a fat-soluble compound with catechol content. Because of this catechol, it is deduced that it shows antioxidant properties (Loussouarn et al., 2017). In addition to its antioxidant property, carnosic acid has various pharmacological effects, including antiinflammatory and anticancer properties (Yang et al., 2017; Shao et al., 2019; Habtemariam, 2023).

It has been clearly demonstrated in the literature that one or more phases of the wound healing process can be modulated by phytochemicals and they can easily penetrate the outer layer of the skin. These properties make natural compounds an important therapeutic category for treating a variety of critical illnesses, including chronic wounds (Khalil et al., 2021). The aim of the present study was to investigate whether phenolic and diterpene compounds have synergistic effects on the oxidative events of ellagic acid and carnosic acid during the healing of diabetic wounds. For this purpose, oxidative effects on days 3 and 7 of wound healing was investigated by applying both topical and oral gavage after making circular excisional wounds in rats with STZ-induced diabetes.

## 2. Materials and methods

### 2.1. Animals

All experimental procedures on the animals were reviewed and approved by the Gazi University Animal Experiments Local Ethics Committee in accordance with local ethical standards and protocols set out in national guidelines (Approval No. G.Ü.ET-20.012). Fifty-four female Wistar albino rats (200–250 g) were obtained from Gazi University Laboratory Animal Breeding and Experimental Research Center (GUDAM) for experiments. The animals were randomly divided into 9 different groups with 6 animals in each group (Table 1). Each animal was housed in a separate cage in an environment lit according to the daylight cycle and was fed ad libitum throughout the experiment.

### 2.2. Preparation of diabetic rats

Streptozotocin (STZ) (Sigma-Aldrich, USA) was used to establish the diabetes model in the animals. STZ was freshly prepared in sodium citrate buffer (0.1 mol/L, pH 4.5); a single dose was administered intraperitoneally. Blood glucose levels of all animals were measured by glucometer (Accu-Chek Performa Nano, Roche Diagnostics, Mannheim, Germany) 72 h after administration, and animals with a value above 250 mg/dL were considered diabetic (Kaltalioglu et al., 2020).

### 2.3. Creating the wound model

The animals prepared for the experiment were given ketamine HCl (50 mg/kg) and xylazine HCl (5 mg/kg) injections into their muscles. This made them deeply anesthetized. The dorsum of the rat was shaved and cleaned to ensure sterility. Six full-thickness excisional skin wounds were created in the animals, except for in the control group, using a 6-mm punch (Acu-Punch; Acuderm, Fort Lauderdale, FL, USA).

### 2.4. Preparation of the ellagic acid and carnosic acid gel formulation

A gel formulation containing ellagic acid and carnosic acid was prepared in the laboratories of Gazi University Faculty of Pharmacy, Department of Pharmaceutical Technology. While preparing the topical gel, 2% Carbopol 974P was used. It was dispersed in 98 g of distilled water containing carnosic acid and stirred at 800 rpm for 60 min. A transparent gel was formed by adding 10% NaOH solution dropwise to this mixture. The solubility of ellagic acid increases with NaOH and is pH dependent. Therefore, this solution contains ellagic acid (Kim et al., 2010). The dose of ellagic acid and carnosic acid was adjusted to 10 mg/kg. After being left for 24 h at +4 °C to remove air bubbles that formed in the gel, it was sterilized under UV and made ready for topical in vivo application. The gel formulation containing ellagic acid and carnosic acid was laid in a thin layer in a petri dish and sterilized in a laminar flow cabinet (Euroflow Class IIA, Clean Air Techniek, Woerden, Netherlands) under a UV lamp (254 nm) for 2 h. In addition, all pharmaceutical characterization studies of the prepared gel formulation were conducted before and after sterilization (viscosity, flow, pH, structure profile analysis, etc.).

### 2.5. Study design

**Figure 1:**
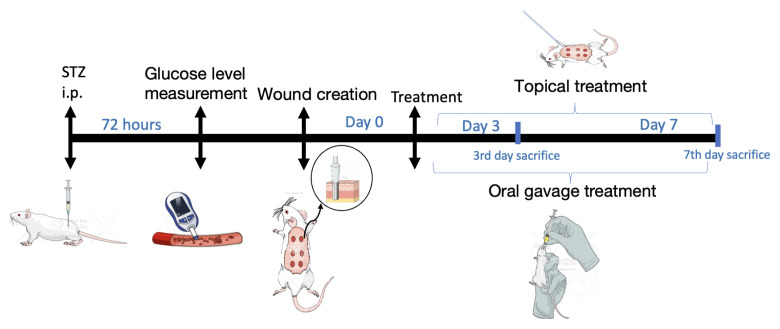
The experimental timeline schematic representation (the experimental overview was created with Mindthegraph.com, accessed on 30 August 2023).

In order to observe the effects of ellagic acid and carnosic acid in combination on diabetic wounds, after injecting healthy rats with STZ (60 mg/kg) those with blood glucose levels of 250 mg/dL were selected. Excisional wounds were created on the selected rats using a punch biopsy. The wounds were treated with a combination of ellagic acid and carnosic acid, both topically and orally, administered on days 3 and 7 (Figure 1).

### 2.6. Wound area and wound contraction

The evaluation of wound areas and assessment of healing progress were expertly executed using the software ImageJ (NIH, Bethesda, MD, USA). Closure of the wound was measured by computing the decrease in the initial wound size as follows:


[(Wound Area on day 0-Wound Area on day 3 or 7)/Wound Area on day 0]×100.

The parameters used to define the wounds were the area and the rate of contraction (Kaltalioglu, 2023).

### 2.7. Determination of histopathological effects on wound healing

Rat tissue samples were fixed in 10% buffered formalin for 12 h and followed up with an automatic tissue tracking device (Leica ASP300, Newcastle, UK). The tissues were embedded in paraffin (Leica Eg1150 embedding device, Newcastle, UK) and 1 block was obtained for each case. Two 3-μm-thick sections (Leica 2125Rt) were taken from these blocks for each case. One section was stained with routine hematoxylin and eosin and the other with Masson’s trichrome (Biognost brand Medjugorska 59, Zagreb, Croatia). The preparations were examined by light microscopy (Olympus brand Cx4, Japan).

### 2.8. Biochemical parameters

#### 2.8.1. Determination of MDA levels

The content of lipid peroxidation products, substances that react with thiobarbituric acid reactive substances (TBARS), is an indicator, and lipid peroxidation is quantified by determination of the formation of malondialdehyde (MDA). The scar tissue was weighed and homogenized in 150 mM KCl (1:9). It was mixed with cold 15% TCA to precipitate the protein in the scar tissue samples. It was centrifuged and the resulting supernatant was taken and 0.67% TBA and 1% BHT (95% ethanol) were added and reacted by double boiler method in a boiling water bath for 10 min. After cooling, the absorbance was read spectrophotometrically at 535 nm. MDA concentration in tissues was calculated as nmol/g tissue (Casini et al., 1986).

#### 2.8.2. Determination of GSH levels

The glutathione (GSH) level in wound tissue was evaluated using the Ellman method (Aykac et al., 1985). Tissue samples were homogenized and deproteinized. After centrifugation, the supernatant, 2 mL of 0.3 M disodium hydrogen phosphate, and 0.2 mL of dithiobisnitrobenzoate solution (0.4 mg/mL 1% sodium citrate) were added. The absorbance of all samples was measured at 412 nm. Tissue GSH levels were calculated as μmol/g tissue.

#### 2.8.3. Determination of NOx levels

The nitric oxide (NOx) concentration in the wound tissues was determined by the Griess method. The wound tissues were homogenized and then centrifuged. The supernatant was taken and VCl_3_ was added to reduce the nitrate in the environment to nitrite and the resulting mixture was incubated at 37 °C for 30 min. Then sodium phosphate buffer and Griess I+II reagents mixed in equal amounts were added followed by incubation at 37 °C for 10 min. The absorbance of the samples was measured at 540 nm (Miranda et al., 2001).

#### 2.8.4. Determination of protein carbonyl levels

Protein carbonyl (PC) content was studied according to the method described by Reznick and Packer (1994). Tissues were homogenized. In order to detect the PC content, two test tubes, one with DNPH and one without DNPH, were prepared. Homogenates were added to the tubes and then DNPH was added to the tube with DNPH and HCl to the tube without DNPH. The tubes, which were vortexed every 15 min, were left at room temperature for 1 h. Next, 20% TCA was added to both tubes and they were centrifuged, the supernatant was discarded, and the pellet continued to work. After 10% TCA was added, the tubes were centrifuged, the supernatant was discarded, and the pellet continued to work. Ethanol/ethyl acetate was added to the tubes, and the supernatant was discarded from the tubes, which were centrifuged, and the pellet continued to work. This process was performed 3 times. Guanidine HCl was added to the tubes followed by vortexing for 10 min; next, the samples were left for 10 min to dissolve and then the absorbance of all samples was measured at 370 nm and 280 nm against guanidine HCl.

#### 2.8.5. Determination of AOPP levels

The detection of advanced oxidation protein products (AOPPs) in serum was determined by ELISA. A commercial kit (Cloud-Clone commercial ELISA kit) was used to determine the amounts of AOPPs in serum samples. Serum AOPP levels were calculated as ng/mL.

#### 2.8.6. Determination of collagen levels

The modified Lowry method was performed for the determination of collagen in wound tissues. Tissue samples were homogenized and the supernatant was taken. Solution A was added followed by incubation at 50 °C for 20 min. After cooling to room temperature, B solution was added. It was incubated for 10 min at room temperature. Then Folin solution was added followed by vortexing. It was incubated at 50 °C for 10 min. It was cooled to room temperature and all samples’ absorbance was measured at 650 nm (Komsa-Penkova et al., 1996).

#### 2.8.7. Determination of MMP-2 and MMP-9

Detection of matrix metalloproteinase 2 (MMP-2) and matrix metalloproteinase 9 (MMP-9) in wound tissues was achieved using ELISA. Wound tissues were weighed and homogenized with lysis buffer (1:50). The homogenates were centrifuged at 3000 × *g* for 5 min and the supernatants were measured using a commercial kit (Cloud-Clone (USA) MMP2 and MMP9 Rat ELISA Kit). Tissue MMP-2 and MMP-9 levels were calculated as ng/mL.

### 2.9. Statistical analyses

The statistical analysis was conducted using GraphPad Prism 8.4.2. The results were presented as mean ± SD. The groups were compared using one-way ANOVA and p-values ≤0.05 were considered significant.

## 3. Results

### 3.1. Wound healing

**Figure 2:**
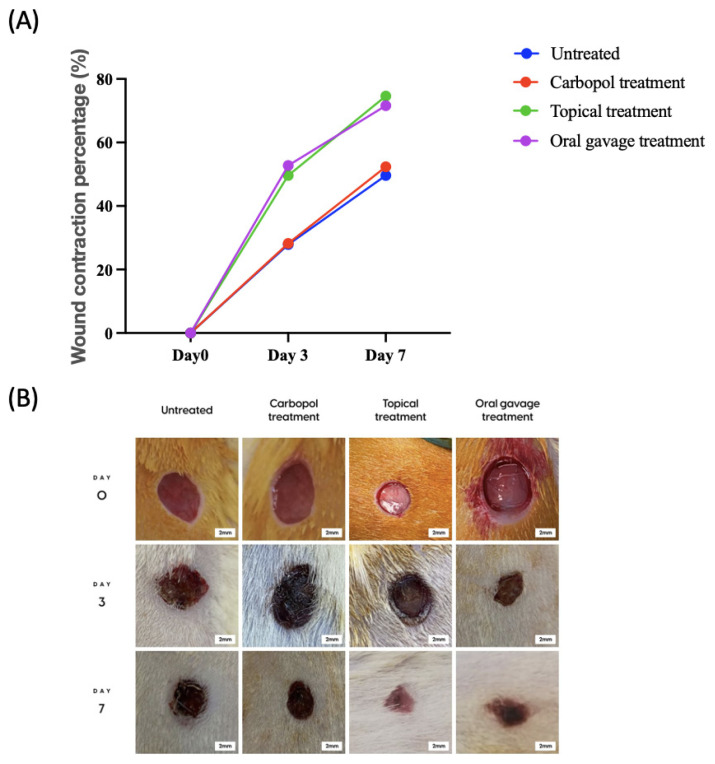
A visual representation of the change in wound contraction rate for all groups (A). Photographs were collected on days 0, 3, and 7 after the full-thickness excisional wound model was created (B).

The line graph in Figure 2A displays the wound contraction percentages, while comparative photographs of wound healing by groups and days are presented in Figure 2B. The day 3 and day 7 groups were compared to the untreated day 0, and a significant reduction in wound areas was detected (p < 0.05). When the groups were evaluated on days 3 and 7, a significant reduction in wound areas was observed on day 7 (p < 0.05). Comparing the untreated groups on the same day with the other groups, a remarkable decrease in the wound area was observed (p < 0.05) (Table 2).

### 3.2. Histopathological assessment

Histology parameters were calculated as in Gupta and Kumar (2015) and van de Vyver et al. (2021) (Table 3).

**Figure 3:**
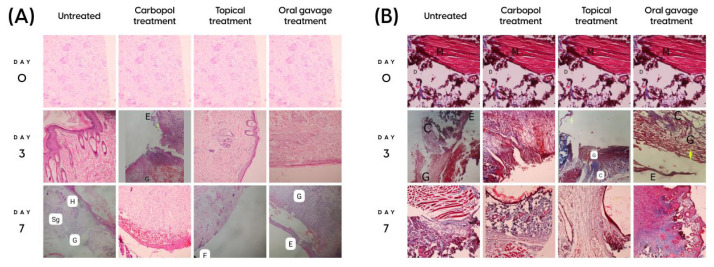
Microscopic images of hematoxylin and eosin in all groups (A). Microscopic images of Masson’s trichrome in all groups (B) C: collagen D: dermis, E; epithelialization G; granulation tissue, H; hairy cell, M: muscle, Sg; fatty gland.

No histopathologic findings were observed in the control group. When the sections of the untreated groups were examined on days 3 and 7, it was observed that reepithelialization was not completed; granulation tissue was underdeveloped; white adipose tissue, hair follicles, and sebaceous glands, which were the signs of the remodeling stage, were not formed; and dermis healing was still in the early stage. In the Carbopol treated groups (both day 3 and day 7), epithelialization was at minimal levels and dermis healing was not observed. Inflammation was noticeable in both groups. In the topical treatment group on day 3, epithelialization was partially completed. Granulation tissue had started to form but there was no evidence of remodeling. In the topical treatment group on day 7, epithelialization was partially complete and subepithelial collagen bundles and hair follicles were seen. In the oral gavage treatment group on day 3, epithelialization was completed. In addition, granulation tissue was thin and the remodeling phase had started. In the oral gavage treatment group on day 7, reepithelialization was completed. Granulation tissue, collagen bundles, hair follicles, and dermal adipose tissue were observed (Table 4; Figure 3).

### 3.3. Biochemical parameters

#### 3.3.1. Wound tissue MDA levels

There was a noteworthy reduction in MDA levels observed in both the treated and untreated groups as compared to the control group (p < 0.05). The Carbopol groups exhibited a significant increase in MDA levels in wound tissue on days 3 and 7 postwounding compared to the untreated groups (p < 0.05).

The wound tissue MDA levels were significantly higher on day 7 compared to day 3 in the untreated group (p < 0.05). The untreated groups were compared with the groups that received topical and oral gavage treatment and a significant decrease was found in the MDA levels of the treated groups (p < 0.05).

**Figure 4:**
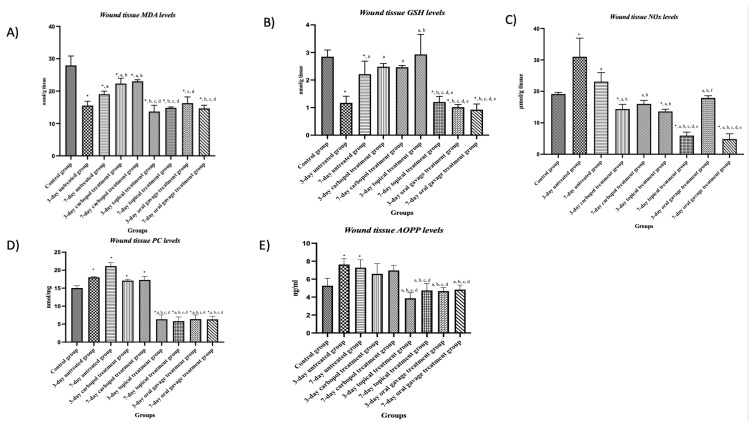
Graphs showing changes in diabetic scar tissue MDA (A), GSH (B), NOx (C), PC (D), and AOPP (E) level in all groups. Values are expressed as mean ± SD. p^*^: compared with the control group, p^a^: compared with the untreated group on day 3, p^b^: compared with the untreated group on day 7, p^c^: compared with the Carbopol treatment group on day 3, p^d^: compared with the Carbopol treatment group on day 7, p^e^: compared with the topical treatment group on day 3, p^f^: compared with the topical treatment group on day 7, p^g^: compared with the oral gavage treatment group on day 3, p < 0.05.

The treatment groups were compared among themselves on day 3 and MDA levels of the topical treated group were significantly lower than those of the oral gavage treatment group (p < 0.05). The treatment groups were also compared among themselves on day 7; there was no significant difference between the topical and oral gavage treatment groups (p > 0.05) (Figure 4A; Table 5).

#### 3.3.2. Wound tissue GSH levels

Comparisons were made between the groups and significant differences in GSH levels were determined between the control group and the other groups (p < 0.05). The GSH levels indicated a significant decrease on day 7 compared to on day 3 in the untreated group (p < 0.05). Upon comparing the Carbopol groups on day 3 to the same day of the other groups, a significant increase in the GSH was observed (p < 0.05). The untreated group and the treated groups were compared on day 3 and a substantial increase was observed in the GSH levels in the topical treated group, while significantly decreased GSH levels were found in the oral gavage treated groups (p < 0.05). The application groups were compared with each other on day 7 and a considerable decrease was found in the GSH levels of both the topical treated and oral gavage treated groups compared to the untreated group (p < 0.05). The topical treatment group was evaluated within itself and it was noted that the GSH levels displayed a significant decrease on day 7 compared to on day 3 (p < 0.05). With topical application, wound tissue GSH levels were significantly increased on day 3 of wound healing when compared with all groups (p < 0.05) (Figure 4B; Table 5).

#### 3.3.3. Wound tissue NOx levels

The control and treated groups were compared and a significant decrease in NOx levels was observed in the treated groups (p < 0.05). The untreated group was evaluated within itself, it was discovered that the NOx levels were decreased on day 7 compared to on day 3 (p<0.05). The groups were compared among themselves on day 3 and a significant decrease was found in the treated groups compared to the untreated group (p > 0.05). When the treated groups were compared on day 7 to the same day of the untreated group, a significant decrease was identified in the NOx levels (p < 0.05). The NOx levels on day 7 in the oral gavage treated group were significantly lower than those of the other treated groups (p < 0.05) (Figure 4C; Table 5).

#### 3.3.4. Wound tissue PC levels

The control group and experimental groups were compared and it was observed that there was a significant difference between all groups (p < 0.05). The groups were compared with each other on day 3 and a significant difference was observed between the untreated and treated groups, with a significant decrease in the scar tissue protein carbonyl levels of the treated groups (p < 0.05). When the untreated group was compared with the topical and oral gavage treated groups on day 7, a significant decrease was identified in the PC levels of the treated groups (p < 0.05) (Figure 4D; Table 5).

#### 3.3.5. Serum AOPP levels

The control group was compared with the other groups and a significant difference was observed between them and the untreated groups (p < 0.05). The treated groups showed decreased serum AOPP levels compared to the untreated groups on days 3 and 7 (p < 0.05). When the treated groups were evaluated among themselves, no significant difference was detected (p > 0.05) (Figure 4E; Table 5).

#### 3.3.6. Wound tissue collagen levels

A significant decrease in wound tissue collagen levels was observed in the treated and untreated groups compared to the control group (p < 0.05). Considering the treated and untreated groups, it was observed that the collagen levels of the treated group increased significantly on days 3 and 7 compared to those of the untreated group (p < 0.05).

When the topical treated group was compared to the oral gavage treated group on day 3, a significant increase was identified in collagen levels (p < 0.05). When the same comparison was performed on day 7, a significant increase was again identified in collagen levels (p < 0.05).

**Figure 5:**
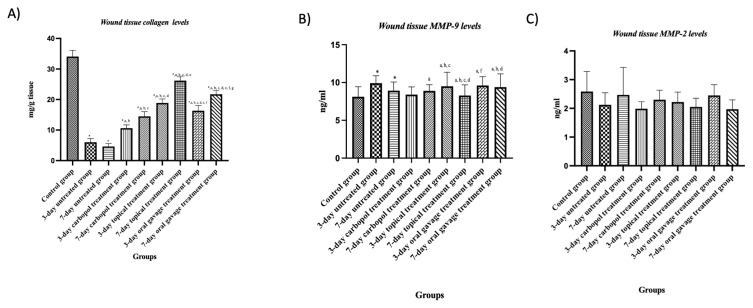
Graphs showing changes in diabetic scar tissue collagen (A), MMP-2 (B), and MMP-9 (C) level in all groups. Values are expressed as mean ± SD. p^*^: compared with the control group, p^a^: compared with the untreated group on day 3, p^b^: compared with the untreated group on day 7, p^c^: compared with the Carbopol treatment group on day 3, p^d^: compared with the Carbopol treatment group on day 7, p^e^: compared with the topical treatment group on day 3, p^f^: compared with the topical treatment group on day 7, p^g^: compared with the oral gavage treatment group on day 3, p < 0.05.

When the topical treated group was evaluated within itself, it was detected that the collagen levels displayed a significant decrease on day 7 compared to those on day 3 (p < 0.05). The oral gavage treated groups were compared with each other and a significant difference was observed between days 3 and 7, and wound tissue collagen levels were increased on day 7 (p < 0.05) (Figure 5A; Table 6).

#### 3.3.7. Wound tissue MMP-2 levels

In terms of MMP-2 levels, the values of days 3 and 7 were compared both among the groups and within the groups and no significant difference was found (p > 0.05) (Figure 5B; Table 6).

#### 3.3.8. Wound tissue MMP-9 levels

The control and untreated groups were compared and a significant increase in MMP-9 levels was found in the untreated group (p < 0.05).

The untreated group was compared with the treated groups on day 3 and a significant decrease was observed in the MMP-9 levels of the topical and oral gavage treated groups (p < 0.05). When the untreated group was compared with the topical and oral gavage treated groups on day 7, a significant decrease was determined in the MMP-9 levels of the topical and oral gavage treated groups (p < 0.05)

When the treated groups were evaluated within themselves, no significant difference was detected in terms of MMP-9 levels between days 3 and 7 of topical or oral gavage treatment (p < 0.05) (Figure 5C; Table 6).

## 4. Discussion

Acute wound healing occurs with the regular progression of hemostasis, inflammation, proliferation, and remodeling stages. Disruption of free radical and antioxidant balance in diabetes leads to the disrupting of different phases of wound healing and delayed healing (Guo and Dipietro, 2010). Following the developing technology, the tendency towards traditionally used plants has increased and attempts are made to explain their mechanisms of action by analyzing their contents. Recently, the use of components with high potential effects such as polyphenols has become widespread in content analysis rather than plant extracts. We investigated the effects of two antioxidants, ellagic acid and carnosic acid, in combination by applications of both topical and oral gavage on different days of diabetic wound healing.

Wound contraction, an indicator of the onset of epithelialization, involves the migration of epithelial cells from the wound edge toward the wound bed. It is also a vital indicator of the onset of wound healing. Previous studies have shown a delay in wound closure in diabetic wounds (Blakytny and Jude, 2006; Mo et al., 2014). Regarding our research, in the treatment groups in which we applied combinations of ellagic acid and carnosic acid both topically and orally, wound areas decreased significantly on both treatment days compared to the untreated groups. Normal skin tissue consists of the epidermis, containing multilayered squamous epithelium, and the dermis, containing collagen fibers and sebaceous glands surrounding hair follicles. The epidermis and dermis layers that are disrupted by wound formation are expected to return to their previous form in healthy wound healing. In our study, epithelialization was incomplete in the untreated groups and no evidence of remodeling such as deposition of collagen fibers or formation of hair follicles was found. In the topically treated groups, epithelialization was partially completed and the remodeling phase was observed. In the oral gavage treated groups, epithelialization was completed and the remodeling phase was supported by histopathological findings. Thus, it can be suggested that both applications contribute to the diabetic wound healing process.

Poor and delayed wound healing is a hallmark of diabetes. Increased glucose levels in diabetes cause oxidative stress. MDA, which occurs as the product of lipid peroxidation, is among the markers of oxidative stress and it is known that MDA levels increase with diabetes (Matteucci and Giampietro, 2000; Gökşen et al., 2017). Evidence suggests that ellagic acid can function as a potent antioxidant due to its lipophilic properties and ability to scavenge peroxyl radicals, making it a promising candidate for chain-breaking antioxidants (Priyadarsini et al., 2002). Al-Obaidi et al. (2014) discovered that ellagic acid, a natural compound, can prevent bone loss in diabetic rats caused by tooth extraction. The research revealed that the antioxidant status of the gingival tissue in diabetic rats improved after being treated with ellagic acid and this resulted in decreased MDA levels, which is an indicator of oxidative stress (Al-Obaidi et al., 2014). Carnosic acid is a lipophilic antioxidant, scavenging singlet oxygen, hydroxyl radicals, and lipid peroxyl radicals and thus preventing lipid peroxidation and the breakdown of biological membranes (Mirza et al., 2023). Aruoma et al. (1992) found that carnosic acid could potentially inhibit lipid peroxidation in microsomal and liposomal systems. In our study, the MDA level in the untreated group was higher on day 7 compared to that on day 3. In both the topical and oral gavage treated groups, MDA levels were lower on day 7. Although there was no significant difference between the topical and oral gavage treatments on day 7, the MDA levels of the topical group were lower than those of the oral gavage group on day 3. In parallel with the literature, the effects of ellagic acid and carnosic acid on free radicals showed a cumulative effect and a protective effect against lipid peroxidation by reducing wound tissue MDA levels due to oxidative stress caused by diabetes.

The GSH redox cycle plays a significant role in endogenous antioxidant defense mechanisms. Total GSH and associated GSH enzymes are crucial biochemical protective systems, as opposed to causing damage in the case of free oxygen radicals (Adamson et al., 1996). The increase in the level of reactive oxygen species in diabetic wounds leads to the consumption of some well-known antioxidant molecules such as GSH (Kopal et al., 2007). In the topical treatment group on day 3, significant increases in GSH values were observed in wound tissues. On the other hand, a decrease in GSH values was detected with topical and oral gavage applications on day 7. Khanduja et al. (2006) applied ellagic acid to normal human peripheral blood mononuclear cells and showed that it inhibited the formation of superoxide anions and hydroxyl radicals in the cells both enzymatically and nonenzymatically and decreased the depletion of GSH and the percentage of cell survival. Sahu et al. (2011) investigated the therapeutic effects of CA on diabetic nephropathy and found that carnosic acid caused a significant increase in GSH levels both before and after treatment. According to our study, while the combination of ellagic acid and carnosic acid did not have an impact on GSH levels in wound tissue, we observed a decrease in MDA values. This suggests that the phenolic compounds used may be linked to the ability to scavenge free radicals.

Including many essential biological functions, nitric oxide (NO) is a short-lived free radical. NO is a molecule that modulates wound healing, such as collagen formation, cell proliferation, and wound contraction (Witte and Barbul, 2002). Low concentrations of NO in the tissue have a protective effect, but high concentrations show a cytotoxic effect (Habib and Ali, 2011). In addition to its contribution to wound healing, like all free radicals it can cause cellular damage when produced at high levels. It is known that NO production increases with increased leukocyte activation in the inflammation phase of wound healing. It is well known that during the inflammation phase of wound healing, leukocyte activation leads to increased production of NO (Uzun et al., 2023). When we evaluated diabetic wound tissue on day 3, we found a significant decrease in NOx levels in the topical and oral gavage treatment groups. We also detected a reduction in NOx levels of topical and oral gavage treatment groups on day 7. When evaluated according to the application methods, it was found that the NOx level of the oral gavage treatment group on day 7 was lower. Priyadarsini et al. (2002) observed that ellagic acid scavenges hydroxyl radicals and NO and has an effect comparable to that of many well-known antioxidants such as vitamin E and vitamin C. Umesalma and Sudhandiran (2010) reported that ellagic acid inhibits NO release. Kuo et al. (2011) showed that carnosic acid significantly suppressed the expression of NO. Xiang et al. (2013) reported that carnosic acid probably prevents the cytotoxic effects of free oxygen radicals, NO, and cytokines. Consistent with the literature, our study found that the combination of ellagic acid and carnosic acid suppressed NOx levels in diabetic wound tissue.

Through the process of wound healing, proteins are the primary molecules liable for the growth of tissue, renewal, and repair. In wound repair, proteins play an essential role in RNA and DNA synthesis, epidermal growth and keratinization, and collagen production (Dryden et al., 2013). Proteins involved in RNA and DNA synthesis, collagen production, epidermal growth, and keratinization significantly affect the wound healing process (Dryden et al., 2013). Increased reactive oxygen species in diabetes leads to protein oxidative damage and peroxidation of membrane lipids (Griesmacher et al., 1995). Another biomarker useful for evaluating oxidative stress is protein carbonyl content. Increased free radicals in the tissue cause modifications in amino acids/proteins and the formation of oxidized protein by-products. Increased reactive carbonyl groups in protein oxidation indicate protein dysfunction as well as oxidative stress (Dalle-Donne et al., 2003). Prolonged exposure of proteins to reactive molecules also leads to the formation of AOPPs (Cheng et al., 2013). PC and AOPP levels were evaluated together in our study and it was observed that PC and AOPP values were lower than the diabetic control group and untreated groups in both treatment periods in both the topical and oral gavage treated groups. In line with the results we obtained, it is thought that the phenolic compounds used have a protective effect on proteins via reactive oxygen species. Stojiljković et al. (2019) found that ellagic acid decreased PC and AOPP levels. Xiang et al. (2013) found that carnosic acid has a protective effect against protein damage caused by reactive oxygen species and reactive nitrogen species. Regarding our research, we found that the combination of ellagic acid and carnosic acid was effective in protecting proteins, whether applied both topically and orally. Ellagic acid and carnosic acid have not been used together in any studies found in the literature, making our study results noteworthy.

Collagen, a component of the extracellular matrix, is a protein that plays a crucial role in wound healing. Compared to the intact and well-organized collagen fibrils in nondiabetic skin, collagen fibrils in diabetic skin are fragmented and disorganized and tensile strength and strain strength are known to increase, and this situation negatively affects diabetic wound healing (Kaltalioglu et al., 2020). Collagen plays an active role in all wound healing processes and its level should be kept in balance for the completion of healthy wound healing (Mathew-Steiner et al., 2021).

The enzymes responsible for collagen production and degradation are MMPs. MMP-2 and MMP-9, which are members of the MMP family that play an active role in wound healing, are involved in cell growth, cell migration, inflammation, and angiogenesis, especially during wound healing (Krejner and Grzela, 2015; Kandhwal et al., 2022). MMP activity is beneficial in normal wound healing, but MMP overexpression in chronic wounds is thought to lead to impairment in extracellular matrix compounds and tissue function. Therefore, it is important to maintain the balance of MMPs and their inhibitors in scar tissue (Chung et al., 2017). Ellagic acid is insoluble at pH 7 but remains soluble at physiological pH. Ellagic acid at pH 7 chelates divalent cations (such as Zn^+2^, Ca^+2^, and Fe^+2^), enhancing its ability to inactivate MMPs such as MMP-1, MMP-2, and MMP-9 (Losso et al., 2004). Liu et al. (2017) found that ellagic acid gradually reduced MMP-2 and MMP-9 levels in cancer studies. Carnosic acid, a catechol-type electrophilic molecule, activates the Keap1/Nrf2 transcriptional pathway by binding specific cysteine residues on Keap1, protecting against oxidative stress (Satoh et al., 2008). Yu et al. (2008) demonstrated that these inhibitory mechanisms of carnosic acid may interrupt a signaling event involving MMP-9 transcription-mediated activation of NF-kB and, based on this conclusion, CA reduced MMP-9 expression. The combination of ellagic acid and carnosic acid applied in our study shows an increase in collagen levels compared to the untreated group. A meaningful reduction in MMP 9 levels was also observed. Especially on day 7 of topical treatment, collagen levels increased and MMP-9 levels decreased compared to the other groups. During our research, there was no difference between the groups in MMP 2 levels. In line with the information in the literature, our study data show that the combination of ellagic acid and carnosic acid increases collagen synthesis in diabetic wounds and contributes to the establishment of collagen balance by suppressing MMP-9 levels that increase with diabetes. The fact that no change was detected in MMP-2 levels on day 3 or 7 may be because MMP-2 activation was more active in the remodeling phase of wound healing.

Considering that a single antioxidant can limit one or several free radicals, two different antioxidants are thought to be more effective in eliminating multiple oxidants that arise during biochemical reactions. In our research, we examined the effects of two different antioxidant polyphenols against oxidative events that occur during diabetic wound healing. Lipid peroxidation decreased with the cumulative effect of ellagic acid and carnosic acid due to their antioxidant properties and their ability to prevent oxidative damage by using different pathways, and the protective effect against proteins showed its effect both in the inflammation phase and the proliferation phase. Closing wound areas, increasing collagen synthesis, and establishing MMP balance have an important role for healthy wound healing. It is observed that the wound closure rate increases in the groups treated with the combination of ellagic acid and carnosic acid; also it increases collagen levels by inhibiting MMP-9 activation. In line with our results, it can be concluded for the first time that the combined use of ellagic acid and carnosic acid triggers and accelerates wound healing and affects oxidative events in diabetic wound healing. Our study may provide insight for the future research in this area.

## Figures and Tables

**Table 1:** Experimental groups.

GROUPS	PROCEDURE
1st group	Control group (diabetic, no wound created) (n = 6)
2nd group	Diabetic, wound created but untreated, assessed on day 3 (n = 6)
3rd group	Diabetic, wound created but untreated, assessed on day 7 (n = 6)
4th group	Diabetic, wound created, and treated by 2% carbopol 974P on day 3 (n = 6)
5th group	Diabetic, wound created, and treated by 2% carbopol 974P on day 7 (n = 6)
6th group	Diabetic, wound created, and treated topically with 2% carbopol 974P + EA + CA gel on day 3 (n = 6)
7th group	Diabetic, wound created, and treated topically with 2% carbopol 974P + EA + CA gel on day 7 (n = 6)
8th group	Diabetic, wound created, and treated with EA + CA oral gavage on day 3 (n = 6)
9th group	Diabetic, wound created, and treated with EA + CA oral gavage on day 7 (n = 6)

**Table 2:** Wound areas (mm^2^) and the rates of wound contraction of rats (values are expressed as the mean ± SD, p^a^: Compared with the untreated group on day 0 (p < 0.05), p^b^: Compared with intragroup day 3 (p < 0.05), p^c^: Compared with the untreated group on the same day (p < 0.05)).

GROUPS	Postwounding (days)	Wound area	The rates of wound contraction
Untreated	Day 0	32.12 ± 2.45	-
	Day 3	23.17 ± 2.37^a^	% 27.86
	Day 7	16.18 ± 2.40^a, b^	% 49.62
Carbopol treatment	Day 0	30.60 ± 3.24	-
	Day 3	21.96 ± 2.05^a^	% 28.23
	Day 7	14.58 ± 2.26^a, b^	% 52.35
Topical treatment	Day 0	32.53 ± 2.31	-
	Day 3	16.39 ± 1.41^a, c^	% 49.61
	Day 7	8.26 ± 1.30^a, b, c^	% 74.60
Oral gavage treatment	Day 0	31.67 ± 1.99	-
	Day 3	14.96 ± 1.37^a, c^	% 52.76
	Day 7	9.01 ± 1.44^a, b, c^	% 71.58

**Table 3:** Histology scoring system: description of parameters

Parameter	Description	Criteria	Score

**Reepithelization**	Complete	95%–100%	++
	Partial	<95%; >0%	+
	None	0%	−

**Epidermal thickness**	Normal	95%–105%	++
	Hypertrophy	>105%	+
	Hypoplasia	<95%	−

**Keratinization**	Yes	Loosely attached/lost layers	++
		OR thick parakeratotic	
	No	None	−

**Granulation**	Intact dermis	Dermal layer intact no granular infiltrates consistent with healing	++
	Thick	>100 μm	+
	Thin	<100 μm	−

**Remodeling**	Complete	Dermal white adipose tissue	++
		Skin appendages	
		Panniculus carnosus regeneration	
	Partial	✓ Collagen deposition	
		✓ Dermal white adipose tissue	+
	None	No evidence	−

**Scar elevation index**	Normal	95%–105%	++
	Hypertrophied	>105%	+
	Hypoplasia	<95%	−

**Inflammation and necrosis**	None	White blood cells matrix remodeling	−
	Mild		+
	Partial		++
	High		+++

**Table 4:** Scoring of the histopathological findings in the skin sections (ND: Not detected).

Sample (tissue)	Reepithelization	Epidermal thickness	Keratinization	Granulation	Remodeling	Scar elevation index	Inflammation and necrosis
Control diabetic group	ND	ND	ND	++	++	++	−
Untreated group (3 days)	+	−	−	+	−	++	+
Untreated group (7 days)	++	+	++	−	+	++	−
Carbopol treatment group (3 days)	+	−	−	+	−	−	+++
Carbopol treatment group (7 days)	++	+	++	+	+	+	+++
EA+CA topical treatment (3 days)	+	−	−	+	−	−	+++
EA+CA topical treatment (7 days)	+	−	−	++	+	−	++
EA+CA oral gavage treatment (3 days)	++	++	−	+	+	−	+
EA+CA oral gavage treatment (7 days)	++	++	++	+	+	+	+++

**Table 5:** Evaluation of MDA, GSH, NOx, and PC levels in diabetic scar tissue and serum AOPP levels. Values are expressed as mean ± SD. p^*^: compared with the control group, p^a^: compared with the untreated group on day 3, p^b^: compared with the untreated group on day 7, p^c^: compared with the Carbopol treatment group on day 3, p^d^: compared with the Carbopol treatment group on day 7, p^e^: compared with the topical treatment group on day 3, p^f^: compared with the topical treatment group on day 7, p^g^: compared with the oral gavage treatment group on day 3, p < 0.05.

GROUPS	MDA (nmol/g)	GSH (μmol/g)	NOx (μmol/g)	PC (nmol/mg)	AOPP (ng/mL)
**Control group**	27.94 ± 2.90	2.84 ± 0.24	19.14± 0.51	15.01 ±0.60	5.27 ± 0.82
**3-day untreated group**	15.53±1.32^*^	1.17± 0.23^*^	31.00±5.92^*^	18.01± 0.21^*^	7.63± 0.65^*^
**7-day untreated group**	19.03±0.95^*, a^	2.21±0.47 ^*, a^	23.10±2.85^a^	21.14 ± 0.92^*^	7.28 ± 0.87^*^
**3-day carbopol treatment group**	22.32 ± 1.84 ^*, a, b^	2.48 ± 0.11^a^	14.37±1.50^*, a, b^	17.08 ±0.36^*^	6.59 ± 1.13
**7-day carbopol treatment group**	23.04 ± 0.55^*, a, b^	2.46 ±0.06^a^	16 ± 1.13^a, b^	17.28±0.88^*^	6.97 ± 0.57
**3-day topical treatment group**	13.68 ± 1.92^*, b, c, d^	2.92 ± 0.72^a, b^	13.62 ± 0.70^*, a, b^	6.34±1.11^*, a,b,c,d^	3.86± 0.62^a, b, c, d^
**7-day topical treatment group**	14.84 ± 0.26^*, b, c, d^	1.20 ± 0.20^*, b, c, d, e^	5.92 ±1.11^*, a, b, c, d, e,^	5.80±1.08^*, a,b,c,d^	4.74±0.77^a, b, c, d^
**3-day oral gavage treatment group**	16.28 ± 1.93^*, c, d^	0.92 ± 0.20^*, b, c, d, e^	17.91 ± 0.71^a, b, f^	6.41±1.03^*, a,b,c,d^	4.67±0.39^a, b, c, d^
**7-day oral gavage treatment group**	14.64 ± 0.98^*, b, c, d^	1.01 ± 0.09^*, b, c, d, e^	4.81 ± 1.68^*, a, b, c, d, e^	6.30±0.80^*, a,b,c,d^	4.83±0.50^a, b, c, d^

**Table 6:** Evaluation of collagen, MMP2, and MMP9 levels in diabetic scar tissue. Values are expressed as mean ± SD. p^*^: compared with the control group, p^a^: compared with the untreated group on day 3, p^b^: compared with the untreated group on day 7, p^c^: compared with the Carbopol treatment group on day 3, p^d^: compared with the Carbopol treatment group on day 7, p^e^: compared with the topical treatment group on day 3, p^f^: compared with the topical treatment group on day 7, p^g^: compared with the oral gavage treatment group on day 3, p < 0.05.

GROUPS	Collagen (μg/mg)	MMP-2 (ng/mL)	MMP-9 (ng/mL)
**Control group**	34.06 ± 2.05	2.58 ± 0.70	8.47 ± 0.39
**3-day untreated group**	6.01 ± 1.17^*^	2.12 ± 0.42	10.29 ± 0.56^*^
**7-day untreated group**	4.61 ± 0.95^*^	2.64 ± 0.81	9.67 ± 0.29^*^
**3-day carbopol treatment group**	10.62 ± 1.68^*, a,b^	1.98 ± 0.24	9.19 ± 0.42
**7-day carbopol treatment group**	14.48 ± 1.54^*, a,b,c^	2.29 ± 0.33	9.04 ± 0.71^a^
**3-day topical treatment group**	18.85 ± 1.30^*, a,b,c,d^	2.11 ± 0.15	7.98 ± 0.38^a, b, c^
**7-day topical treatment group**	26.19 ± 1.26^*, a,b,c,d,e^	2.02 ± 0.29	7.50 ± 0.71 ^a, b, c, d^
**3-day oral gavage treatment group**	16.33 ± 1.73^*, a,b,c,e,f^	2.45 ± 0.37	8.84 ± 0.53^a, f^
**7-day oral gavage treatment group**	21.72 ± 1.18^*,a,b,c,d,e,f,g^	1.97 ± 0.32	8.12 ± 0.61^a, b, d^
